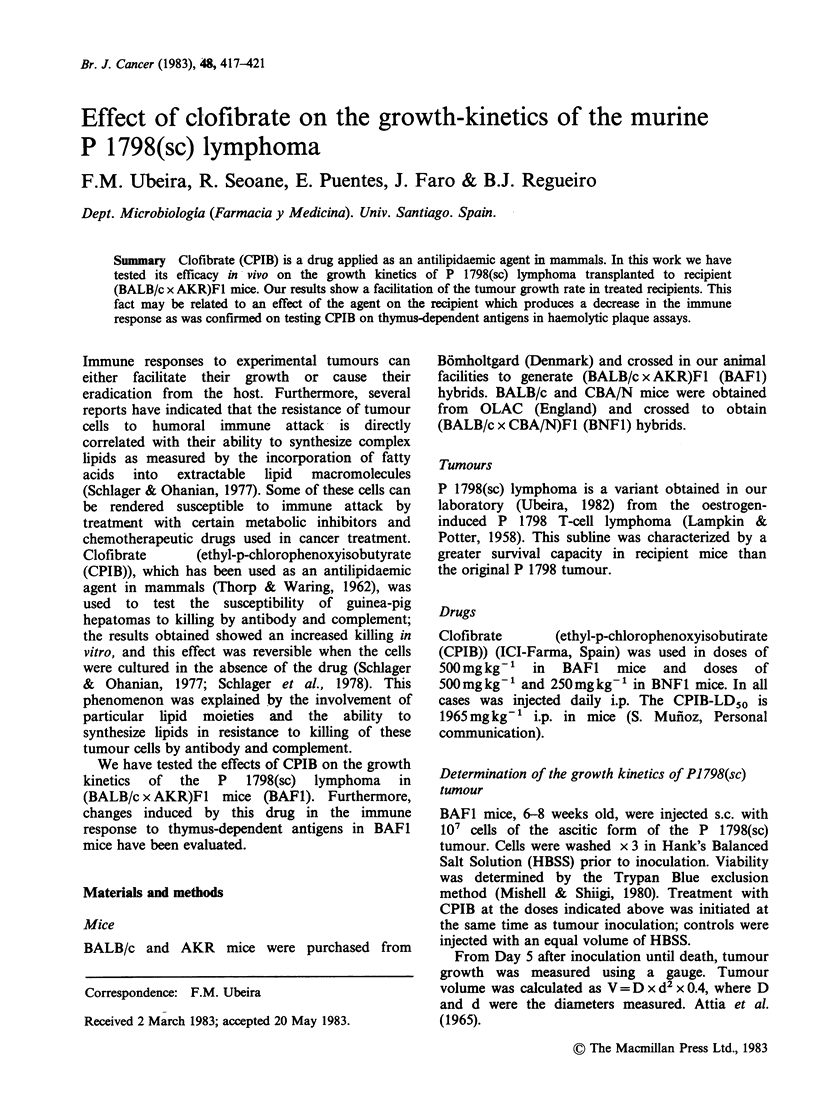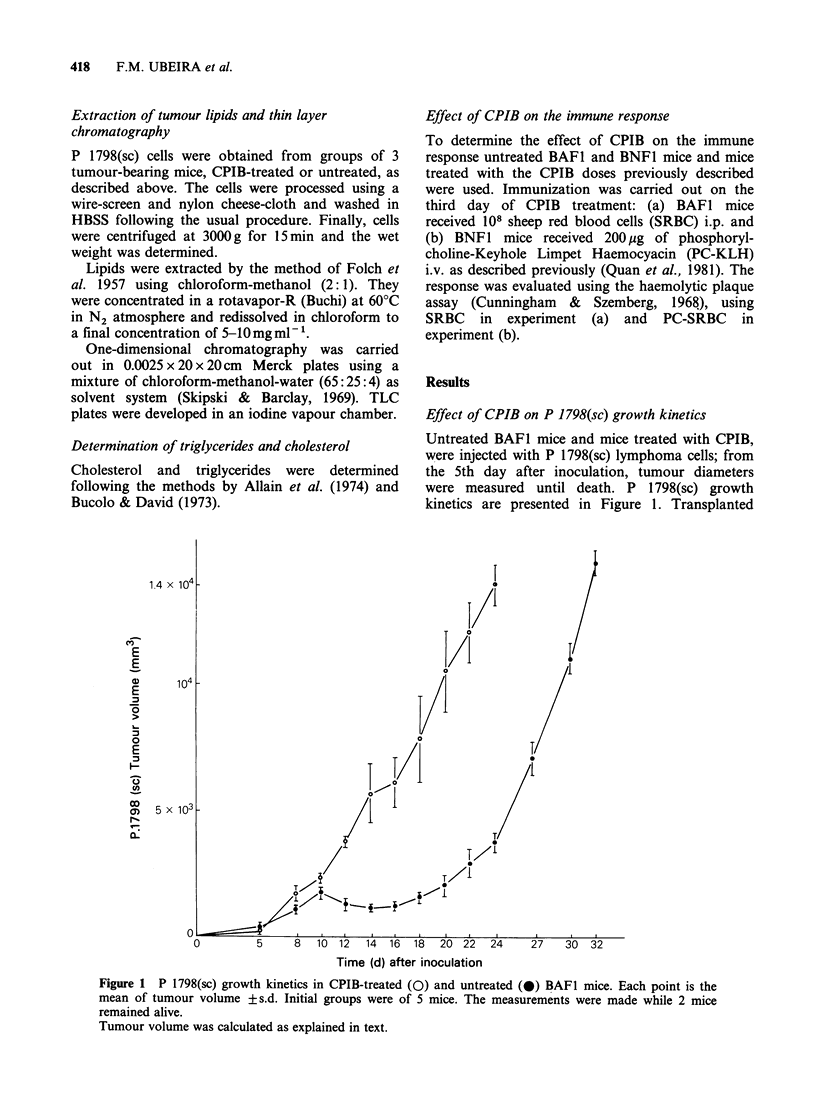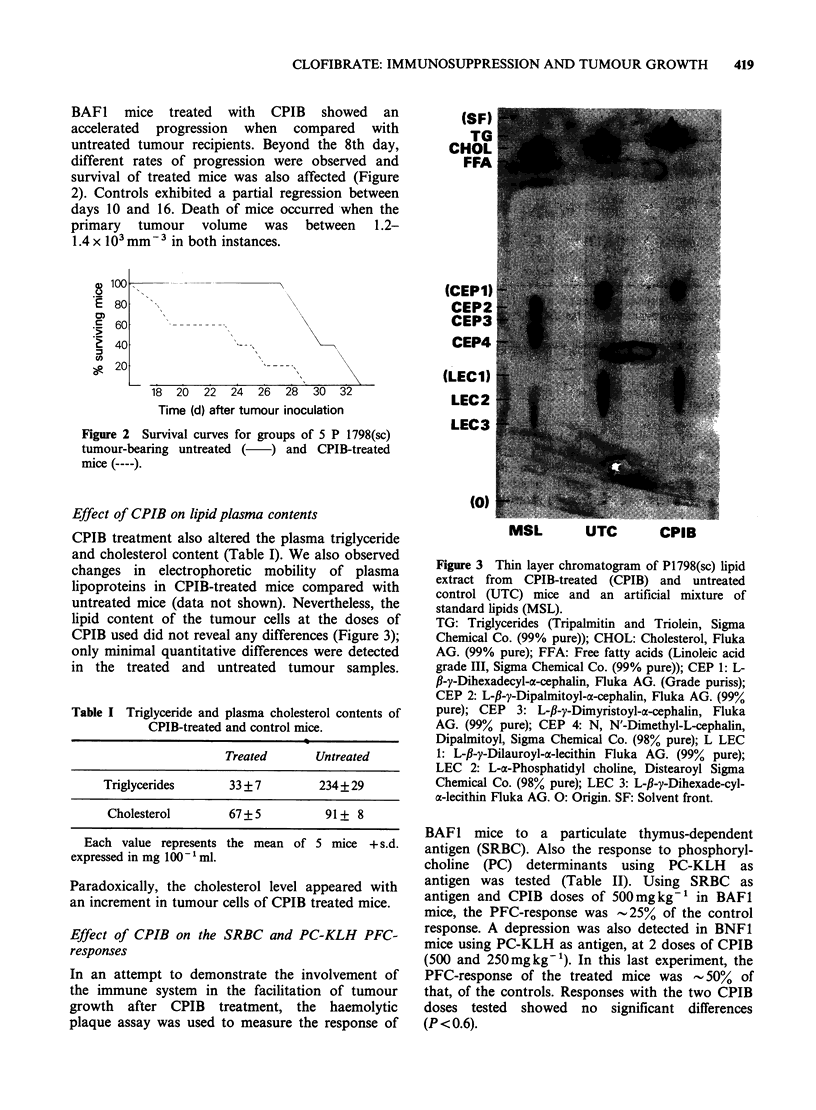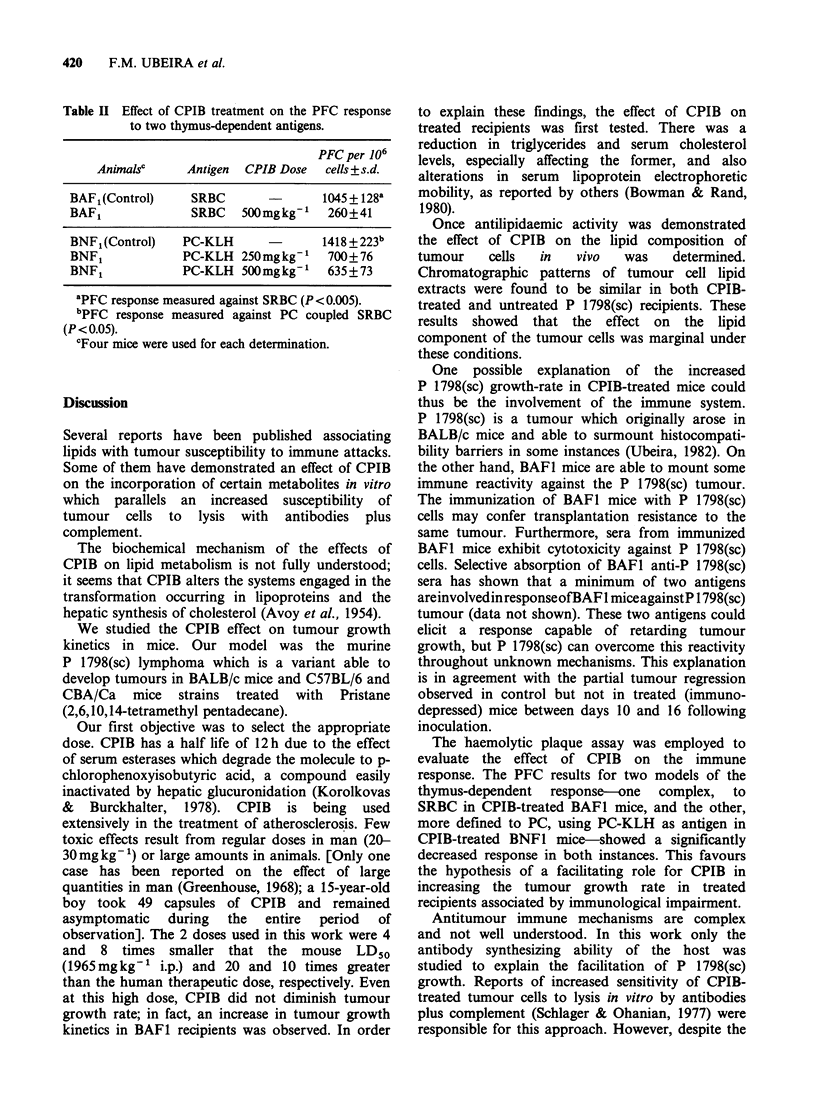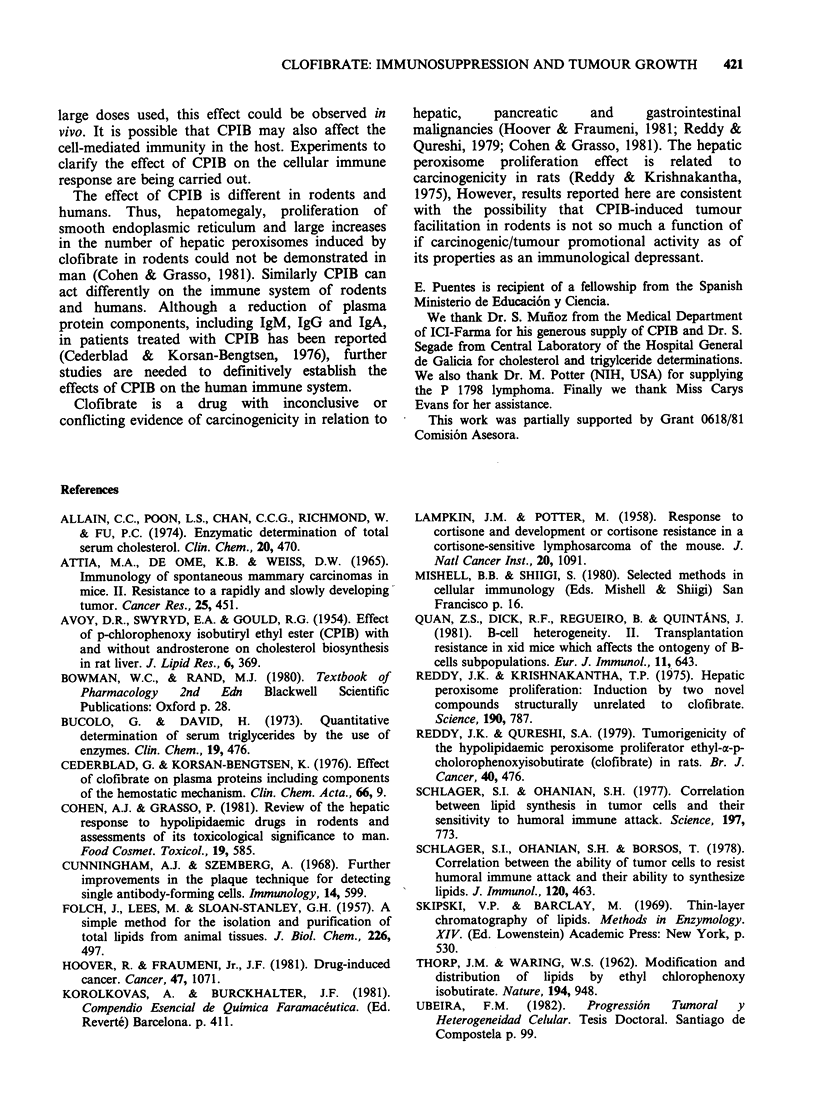# Effect of clofibrate on the growth-kinetics of the murine P 1798(sc) lymphoma.

**DOI:** 10.1038/bjc.1983.207

**Published:** 1983-09

**Authors:** F. M. Ubeira, R. Seoane, E. Puentes, J. Faro, B. J. Regueiro

## Abstract

**Images:**


*Br. J. Cancer* (1983), **48**, 417–421

### Effect of clofibrate on the growth-kinetics of the murine P 1798(sc) lymphoma

F.M. Ubeira, R. Seoane, E. Puentes, J. Faro & B.J. Regueiro

*Dept. Microbiologia (Farmacia y Medicina). Univ. Santiago. Spain.*

**Summary** Clofibrate (CPIB) is a drug applied as an antilipidaemic agent in mammals. In this work we have tested its efficacy *in vivo* on the growth kinetics of P 1798(sc) lymphoma transplanted to recipient (BALB/c x AKR)F1 mice. Our results show a facilitation of the tumour growth rate in treated recipients. This fact may be related to an effect of the agent on the recipient which produces a decrease in the immune response as was confirmed on testing CPIB on thymus-dependent antigens in haemolytic plaque assays.

Immune responses to experimental tumours can either facilitate their growth or cause their eradication from the host. Furthermore, several reports have indicated that the resistance of tumour cells to humoral immune attack is directly correlated with their ability to synthesize complex lipids as measured by the incorporation of fatty acids into extractable lipid macromolecules (Schlager & Ohanian, 1977). Some of these cells can be rendered susceptible to immune attack by treatment with certain metabolic inhibitors and chemotherapeutic drugs used in cancer treatment. Clofibrate (ethyl-p-chlorophenoxyisobutyrate (CPIB)), which has been used as an antilipidaemic agent in mammals (Thorp & Waring, 1962), was used to test the susceptibility of guinea-pig hepatomas to killing by antibody and complement; the results obtained showed an increased killing *in vitro,* and this effect was reversible when the cells were cultured in the absence of the drug (Schlager & Ohanian, 1977; Schlager *et al.,* 1978). This phenomenon was explained by the involvement of particular lipid moieties and the ability to synthesize lipids in resistance to killing of these tumour cells by antibody and complement.

We have tested the effects of CPIB on the growth kinetics of the P 1798(sc) lymphoma in (BALB/c x AKR)F1 mice (BAF1). Furthermore, changes induced by this drug in the immune response to thymus-dependent antigens in BAF1 mice have been evaluated.

### Materials and methods

### *Mice*

BALB/c and AKR mice were purchased from Bömholtgard (Denmark) and crossed in our animal facilities to generate (BALB/c x AKR)F1 (BAF1) hybrids. BALB/c and CBA/N mice were obtained from OLAC (England) and crossed to obtain (BALB/c x CBA/N)F1 (BNF1) hybrids.

### *Tumours*

P 1798(sc) lymphoma is a variant obtained in our laboratory (Ubeira, 1982) from the oestrogen-induced P 1798 T-cell lymphoma (Lampkin & Potter, 1958). This subline was characterized by a greater survival capacity in recipient mice than the original P 1798 tumour.

### *Drugs*

Clofibrate (ethyl-p-chlorophenoxyisobutirate (CPIB)) (ICI-Farma, Spain) was used in doses of $500\,mg\,kg^{-1}$ in BAF1 mice and doses of $500\,mg\,kg^{-1}$ and $250\,mg\,kg^{-1}$ in BNF1 mice. In all cases was injected daily i.p. The CPIB-$LD_{50}$ is $1965\,mg\,kg^{-1}$ i.p. in mice (S. Muñoz, Personal communication).

### *Determination of the growth kinetics of P1798(sc) tumour*

BAF1 mice, 6–8 weeks old, were injected s.c. with $10^7$ cells of the ascitic form of the P 1798(sc) tumour. Cells were washed x 3 in Hank's Balanced Salt Solution (HBSS) prior to inoculation. Viability was determined by the Trypan Blue exclusion method (Mishell & Shiigi, 1980). Treatment with CPIB at the doses indicated above was initiated at the same time as tumour inoculation; controls were injected with an equal volume of HBSS.

From Day 5 after inoculation until death, tumour growth was measured using a gauge. Tumour volume was calculated as $V = D \times d^2 \times 0.4$, where D and d were the diameters measured. Attia *et al.* (1965).

Correspondence: F.M. Ubeira

Received 2 March 1983; accepted 20 May 1983.

© The Macmillan Press Ltd., 1983

*Extraction of tumour lipids and thin layer chromatography*

P 1798(sc) cells were obtained from groups of 3 tumour-bearing mice, CPIB-treated or untreated, as described above. The cells were processed using a wire-screen and nylon cheese-cloth and washed in HBSS following the usual procedure. Finally, cells were centrifuged at 3000 g for 15 min and the wet weight was determined.

Lipids were extracted by the method of Folch *et al.* 1957 using chloroform-methanol (2:1). They were concentrated in a rotavapor-R (Buchi) at 60°C in $N_2$ atmosphere and redissolved in chloroform to a final concentration of 5–10 mg ml$^{-1}$.

One-dimensional chromatography was carried out in 0.0025 x 20 x 20 cm Merck plates using a mixture of chloroform-methanol-water (65:25:4) as solvent system (Skipski & Barclay, 1969). TLC plates were developed in an iodine vapour chamber.

*Determination of triglycerides and cholesterol*

Cholesterol and triglycerides were determined following the methods by Allain *et al.* (1974) and Bucolo & David (1973).

*Effect of CPIB on the immune response*

To determine the effect of CPIB on the immune response untreated BAF1 and BNF1 mice and mice treated with the CPIB doses previously described were used. Immunization was carried out on the third day of CPIB treatment: (a) BAF1 mice received $10^8$ sheep red blood cells (SRBC) i.p. and (b) BNF1 mice received 200 $\mu$g of phosphoryl-choline-Keyhole Limpet Haemocyacin (PC-KLH) i.v. as described previously (Quan *et al.*, 1981). The response was evaluated using the haemolytic plaque assay (Cunningham & Szemberg, 1968), using SRBC in experiment (a) and PC-SRBC in experiment (b).

### Results

*Effect of CPIB on P 1798(sc) growth kinetics*

Untreated BAF1 mice and mice treated with CPIB, were injected with P 1798(sc) lymphoma cells; from the 5th day after inoculation, tumour diameters were measured until death. P 1798(sc) growth kinetics are presented in Figure 1. Transplanted

**Figure 1** P 1798(sc) growth kinetics in CPIB-treated (○) and untreated (●) BAF1 mice. Each point is the mean of tumour volume ±s.d. Initial groups were of 5 mice. The measurements were made while 2 mice remained alive.
Tumour volume was calculated as explained in text.

BAF1 mice treated with CPIB showed an accelerated progression when compared with untreated tumour recipients. Beyond the 8th day, different rates of progression were observed and survival of treated mice was also affected (Figure 2). Controls exhibited a partial regression between days 10 and 16. Death of mice occurred when the primary tumour volume was between 1.2–$1.4 \times 10^3 mm^{-3}$ in both instances.

**Figure 2** Survival curves for groups of 5 P 1798(sc) tumour-bearing untreated (——) and CPIB-treated mice (----).

*Effect of CPIB on lipid plasma contents*

CPIB treatment also altered the plasma triglyceride and cholesterol content (Table I). We also observed changes in electrophoretic mobility of plasma lipoproteins in CPIB-treated mice compared with untreated mice (data not shown). Nevertheless, the lipid content of the tumour cells at the doses of CPIB used did not reveal any differences (Figure 3); only minimal quantitative differences were detected in the treated and untreated tumour samples.

**Table I** Triglyceride and plasma cholesterol contents of CPIB-treated and control mice.

| | *Treated* | *Untreated* |
|---|---|---|
| Triglycerides | 33±7 | 234±29 |
| Cholesterol | 67±5 | 91± 8 |

Each value represents the mean of 5 mice +s.d. expressed in mg $100^{-1}$ ml.

Paradoxically, the cholesterol level appeared with an increment in tumour cells of CPIB treated mice.

*Effect of CPIB on the SRBC and PC-KLH PFC-responses*

In an attempt to demonstrate the involvement of the immune system in the facilitation of tumour growth after CPIB treatment, the haemolytic plaque assay was used to measure the response of BAF1 mice to a particulate thymus-dependent antigen (SRBC). Also the response to phosphorylcholine (PC) determinants using PC-KLH as antigen was tested (Table II). Using SRBC as antigen and CPIB doses of $500 mg kg^{-1}$ in BAF1 mice, the PFC-response was ~25% of the control response. A depression was also detected in BNF1 mice using PC-KLH as antigen, at 2 doses of CPIB (500 and $250 mg kg^{-1}$). In this last experiment, the PFC-response of the treated mice was ~50% of that, of the controls. Responses with the two CPIB doses tested showed no significant differences ($P<0.6$).

**Figure 3** Thin layer chromatogram of P1798(sc) lipid extract from CPIB-treated (CPIB) and untreated control (UTC) mice and an artificial mixture of standard lipids (MSL).
TG: Triglycerides (Tripalmitin and Triolein, Sigma Chemical Co. (99% pure)); CHOL: Cholesterol, Fluka AG. (99% pure); FFA: Free fatty acids (Linoleic acid grade III, Sigma Chemical Co. (99% pure)); CEP 1: L-β-γ-Dihexadecyl-α-cephalin, Fluka AG. (Grade puriss); CEP 2: L-β-γ-Dipalmitoyl-α-cephalin, Fluka AG. (99% pure); CEP 3: L-β-γ-Dimyristoyl-α-cephalin, Fluka AG. (99% pure); CEP 4: N, N'-Dimethyl-L-cephalin, Dipalmitoyl, Sigma Chemical Co. (98% pure); L LEC 1: L-β-γ-Dilauroyl-α-lecithin Fluka AG. (99% pure); LEC 2: L-α-Phosphatidyl choline, Distearoyl Sigma Chemical Co. (98% pure); LEC 3: L-β-γ-Dihexade-cyl-α-lecithin Fluka AG. O: Origin. SF: Solvent front.

**Table II** Effect of CPIB treatment on the PFC response to two thymus-dependent antigens.

| *Animals*[c] | *Antigen* | *CPIB Dose* | *PFC per $10^6$ cells ± s.d.* |
|---|---|---|---|
| $BAF_1$(Control) | SRBC | — | 1045 ± 128[a] |
| $BAF_1$ | SRBC | 500 mg $kg^{-1}$ | 260 ± 41 |
| $BNF_1$(Control) | PC-KLH | — | 1418 ± 223[b] |
| $BNF_1$ | PC-KLH | 250 mg $kg^{-1}$ | 700 ± 76 |
| $BNF_1$ | PC-KLH | 500 mg $kg^{-1}$ | 635 ± 73 |

[a]PFC response measured against SRBC ($P < 0.005$).
[b]PFC response measured against PC coupled SRBC ($P < 0.05$).
[c]Four mice were used for each determination.

### Discussion

Several reports have been published associating lipids with tumour susceptibility to immune attacks. Some of them have demonstrated an effect of CPIB on the incorporation of certain metabolites *in vitro* which parallels an increased susceptibility of tumour cells to lysis with antibodies plus complement.

The biochemical mechanism of the effects of CPIB on lipid metabolism is not fully understood; it seems that CPIB alters the systems engaged in the transformation occurring in lipoproteins and the hepatic synthesis of cholesterol (Avoy *et al.*, 1954).

We studied the CPIB effect on tumour growth kinetics in mice. Our model was the murine P 1798(sc) lymphoma which is a variant able to develop tumours in BALB/c mice and C57BL/6 and CBA/Ca mice strains treated with Pristane (2,6,10,14-tetramethyl pentadecane).

Our first objective was to select the appropriate dose. CPIB has a half life of 12 h due to the effect of serum esterases which degrade the molecule to p-chlorophenoxyisobutyric acid, a compound easily inactivated by hepatic glucuronidation (Korolkovas & Burckhalter, 1978). CPIB is being used extensively in the treatment of atherosclerosis. Few toxic effects result from regular doses in man (20–30 mg $kg^{-1}$) or large amounts in animals. [Only one case has been reported on the effect of large quantities in man (Greenhouse, 1968); a 15-year-old boy took 49 capsules of CPIB and remained asymptomatic during the entire period of observation]. The 2 doses used in this work were 4 and 8 times smaller that the mouse $LD_{50}$ (1965 mg $kg^{-1}$ i.p.) and 20 and 10 times greater than the human therapeutic dose, respectively. Even at this high dose, CPIB did not diminish tumour growth rate; in fact, an increase in tumour growth kinetics in BAF1 recipients was observed. In order to explain these findings, the effect of CPIB on treated recipients was first tested. There was a reduction in triglycerides and serum cholesterol levels, especially affecting the former, and also alterations in serum lipoprotein electrophoretic mobility, as reported by others (Bowman & Rand, 1980).

Once antilipidaemic activity was demonstrated the effect of CPIB on the lipid composition of tumour cells *in vivo* was determined. Chromatographic patterns of tumour cell lipid extracts were found to be similar in both CPIB-treated and untreated P 1798(sc) recipients. These results showed that the effect on the lipid component of the tumour cells was marginal under these conditions.

One possible explanation of the increased P 1798(sc) growth-rate in CPIB-treated mice could thus be the involvement of the immune system. P 1798(sc) is a tumour which originally arose in BALB/c mice and able to surmount histocompatibility barriers in some instances (Ubeira, 1982). On the other hand, BAF1 mice are able to mount some immune reactivity against the P 1798(sc) tumour. The immunization of BAF1 mice with P 1798(sc) cells may confer transplantation resistance to the same tumour. Furthermore, sera from immunized BAF1 mice exhibit cytotoxicity against P 1798(sc) cells. Selective absorption of BAF1 anti-P 1798(sc) sera has shown that a minimum of two antigens areinvolvedinresponseofBAF1miceagainstP1798(sc) tumour (data not shown). These two antigens could elicit a response capable of retarding tumour growth, but P 1798(sc) can overcome this reactivity throughout unknown mechanisms. This explanation is in agreement with the partial tumour regression observed in control but not in treated (immunodepressed) mice between days 10 and 16 following inoculation.

The haemolytic plaque assay was employed to evaluate the effect of CPIB on the immune response. The PFC results for two models of the thymus-dependent response—one complex, to SRBC in CPIB-treated BAF1 mice, and the other, more defined to PC, using PC-KLH as antigen in CPIB-treated BNF1 mice—showed a significantly decreased response in both instances. This favours the hypothesis of a facilitating role for CPIB in increasing the tumour growth rate in treated recipients associated by immunological impairment.

Antitumour immune mechanisms are complex and not well understood. In this work only the antibody synthesizing ability of the host was studied to explain the facilitation of P 1798(sc) growth. Reports of increased sensitivity of CPIB-treated tumour cells to lysis *in vitro* by antibodies plus complement (Schlager & Ohanian, 1977) were responsible for this approach. However, despite the large doses used, this effect could be observed *in vivo*. It is possible that CPIB may also affect the cell-mediated immunity in the host. Experiments to clarify the effect of CPIB on the cellular immune response are being carried out.

The effect of CPIB is different in rodents and humans. Thus, hepatomegaly, proliferation of smooth endoplasmic reticulum and large increases in the number of hepatic peroxisomes induced by clofibrate in rodents could not be demonstrated in man (Cohen & Grasso, 1981). Similarly CPIB can act differently on the immune system of rodents and humans. Although a reduction of plasma protein components, including IgM, IgG and IgA, in patients treated with CPIB has been reported (Cederblad & Korsan-Bengtsen, 1976), further studies are needed to definitively establish the effects of CPIB on the human immune system.

Clofibrate is a drug with inconclusive or conflicting evidence of carcinogenicity in relation to hepatic, pancreatic and gastrointestinal malignancies (Hoover & Fraumeni, 1981; Reddy & Qureshi, 1979; Cohen & Grasso, 1981). The hepatic peroxisome proliferation effect is related to carcinogenicity in rats (Reddy & Krishnakantha, 1975), However, results reported here are consistent with the possibility that CPIB-induced tumour facilitation in rodents is not so much a function of if carcinogenic/tumour promotional activity as of its properties as an immunological depressant.

E. Puentes is recipient of a fellowship from the Spanish Ministerio de Educación y Ciencia.

We thank Dr. S. Muñoz from the Medical Department of ICI-Farma for his generous supply of CPIB and Dr. S. Segade from Central Laboratory of the Hospital General de Galicia for cholesterol and trigylceride determinations. We also thank Dr. M. Potter (NIH, USA) for supplying the P 1798 lymphoma. Finally we thank Miss Carys Evans for her assistance.

This work was partially supported by Grant 0618/81 Comisión Asesora.

**References**

ALLAIN, C.C., POON, L.S., CHAN, C.C.G., RICHMOND, W. & FU, P.C. (1974). Enzymatic determination of total serum cholesterol. *Clin. Chem.*, **20**, 470.

ATTIA, M.A., DE OME, K.B. & WEISS, D.W. (1965). Immunology of spontaneous mammary carcinomas in mice. II. Resistance to a rapidly and slowly developing tumor. *Cancer Res.*, **25**, 451.

AVOY, D.R., SWYRYD, E.A. & GOULD, R.G. (1954). Effect of p-chlorophenoxy isobutiryl ethyl ester (CPIB) with and without androsterone on cholesterol biosynthesis in rat liver. *J. Lipid Res.*, **6**, 369.

BOWMAN, W.C., & RAND, M.J. (1980). *Textbook of Pharmacology 2nd Edn* Blackwell Scientific Publications: Oxford p. 28.

BUCOLO, G. & DAVID, H. (1973). Quantitative determination of serum triglycerides by the use of enzymes. *Clin. Chem.*, **19**, 476.

CEDERBLAD, G. & KORSAN-BENGTSEN, K. (1976). Effect of clofibrate on plasma proteins including components of the hemostatic mechanism. *Clin. Chem. Acta.*, **66**, 9.

COHEN, A.J. & GRASSO, P. (1981). Review of the hepatic response to hypolipidaemic drugs in rodents and assessments of its toxicological significance to man. *Food Cosmet. Toxicol.*, **19**, 585.

CUNNINGHAM, A.J. & SZEMBERG, A. (1968). Further improvements in the plaque technique for detecting single antibody-forming cells. *Immunology*, **14**, 599.

FOLCH, J., LEES, M. & SLOAN-STANLEY, G.H. (1957). A simple method for the isolation and purification of total lipids from animal tissues. *J. Biol. Chem.*, **226**, 497.

HOOVER, R. & FRAUMENI, Jr., J.F. (1981). Drug-induced cancer. *Cancer*, **47**, 1071.

KOROLKOVAS, A. & BURCKHALTER, J.F. (1981). *Compendio Esencial de Quimica Faramacéutica.* (Ed. Reverté) Barcelona. p. 411.

LAMPKIN, J.M. & POTTER, M. (1958). Response to cortisone and development or cortisone resistance in a cortisone-sensitive lymphosarcoma of the mouse. *J. Natl Cancer Inst.*, **20**, 1091.

MISHELL, B.B. & SHIIGI, S. (1980). Selected methods in cellular immunology (Eds. Mishell & Shiigi) San Francisco p. 16.

QUAN, Z.S., DICK, R.F., REGUEIRO, B. & QUINTÁNS, J. (1981). B-cell heterogeneity. II. Transplantation resistance in xid mice which affects the ontogeny of B-cells subpopulations. *Eur. J. Immunol.*, **11**, 643.

REDDY, J.K. & KRISHNAKANTHA, T.P. (1975). Hepatic peroxisome proliferation: Induction by two novel compounds structurally unrelated to clofibrate. *Science*, **190**, 787.

REDDY, J.K. & QURESHI, S.A. (1979). Tumorigenicity of the hypolipidaemic peroxisome proliferator ethyl-α-p-cholorophenoxyisobutirate (clofibrate) in rats. *Br. J. Cancer*, **40**, 476.

SCHLAGER, S.I. & OHANIAN, S.H. (1977). Correlation between lipid synthesis in tumor cells and their sensitivity to humoral immune attack. *Science*, **197**, 773.

SCHLAGER, S.I., OHANIAN, S.H. & BORSOS, T. (1978). Correlation between the ability of tumor cells to resist humoral immune attack and their ability to synthesize lipids. *J. Immunol.*, **120**, 463.

SKIPSKI, V.P. & BARCLAY, M. (1969). Thin-layer chromatography of lipids. *Methods in Enzymology. XIV.* (Ed. Lowenstein) Academic Press: New York, p. 530.

THORP, J.M. & WARING, W.S. (1962). Modification and distribution of lipids by ethyl chlorophenoxy isobutirate. *Nature*, **194**, 948.

UBEIRA, F.M. (1982). *Progressión Tumoral y Heterogeneidad Celular.* Tesis Doctoral. Santiago de Compostela p. 99.